# Second‐Order Topological Insulator in Ferromagnetic Monolayer and Antiferromagnetic Bilayer CrSBr

**DOI:** 10.1002/smsc.202300356

**Published:** 2024-03-15

**Authors:** Zhenzhou Guo, Haoqian Jiang, Lei Jin, Xiaoming Zhang, Guodong Liu, Ying Liu, Xiaotian Wang

**Affiliations:** ^1^ State Key Laboratory of Reliability and Intelligence of Electrical Equipment School of Materials Science and Engineering Hebei University of Technology Tianjin 300130 China; ^2^ Institute for Superconducting and Electronic Materials Faculty of Engineering and Information Sciences University of Wollongong Wollongong 2500 Australia

**Keywords:** 2D, antiferromagnetic, ferromagnetic, fully spin‐polarized corner states, second‐order topological insulators

## Abstract

Second‐order topological insulators (SOTIs) in 2D materials have attracted significant research interest. Recent theoretical predictions suggest that SOTIs can be achievable in 2D magnetic systems, especially within ferromagnetic (FM) materials. Yet, the quest for suitable 2D antiferromagnetic (AFM) materials capable of hosting magnetic SOTIs remains a challenge. Herein, utilizing first‐principles calculations and theoretical analysis, 2D CrSBr is proposed, including monolayer and bilayer forms, as a promising candidate for a magnetic high‐order topological insulator. The monolayer exhibits a FM ground state and features quantized fractional corner charge in its spin‐up channel in the absence of spin–orbital coupling (SOC), yielding fully spin‐polarized corner states. Intriguingly, the bilayer form adopts an AFM ground state while retaining the SOTI properties, with quantized corner charge in both spin channels. Remarkably, the SOTI properties in both monolayer and bilayer structures remain robust against the influence of SOC and symmetry‐breaking perturbations. The work not only identifies a tangible material for realizing 2D magnetic SOTIs, encompassing both FM and AFM phases, but also offers a path to explore the distinctive characteristics of SOTIs merged with magnetism.

## Graphical abstract

This study demonstrates that the second‐order topological insulators phase can be achieved in both ferromagnetic monolayer and antiferromagnetic bilayer CrSBr. Among them, the topological corner states in monolayer CrSBr are exclusively present in the spin‐up channel, giving rise to fully spin‐polarized corner states. Furthermore, these topological corner states exhibit remarkable resilience against the effects of spin–orbit coupling and symmetry‐breaking perturbations.

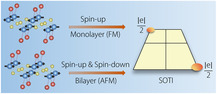

## 1 Introduction

The emergence of topological insulators (TIs) has given rise to a thriving field of research.^[^ ^1^, ^2^, ^3^, ^4^, ^5^, ^6^, ^7^, ^8^, ^9^ ^]^ Traditionally recognized by its defining feature, a TI in *d* spatial dimensions stands apart by possessing an insulating bulk while simultaneously revealing protected, gapless states along its (*d–*1)‐dimensional boundaries. Recently, this paradigm has extended to introduce a novel category of topological phases known as high‐order TIs. In an *n*th‐order TI,^[^ ^10^, ^11^, ^12^, ^13^, ^14^, ^15^, ^16^, ^17^ ^]^ protected gapless states reside at its (*d–n*) boundary, often emerging at the juncture of *n* crystal faces, while the rest of the material remains gapped. For example, a second‐order TI (SOTI) in 2D (3D) has topologically protected states at its 0D corners (1D hinges). To date, high‐order TIs have been predicted in a few 3D materials and several 2D materials. However, possibly due to the complicated magnetic configuration of materials, there are still limited magnetic SOTIs, especially antiferromagnetic (AFM) SOTIs.

The intricate interplay between magnetism and topology has substantially enriched our understanding of various exotic quantum phenomena, including the quantum anomalous Hall effect,^[^ ^18^, ^19^, ^20^ ^]^ fully spin‐polarized topological semimetals,^[^ ^21^, ^22^, ^23^, ^24^ ^]^ and axion TIs.^[^ ^25^, ^26^, ^27^, ^28^ ^]^ However, it is worth noting that the pool of materials capable of realizing high‐order topological phases remains quite limited. To date, only a handful of 2D magnetic SOTIs have been identified, such as bismuthene on a ferromagnetic (FM) EuO (111) surface,^[^ ^29^ ^]^ CrSiTe_3_,^[^ ^30^ ^]^ CrOCl,^[^ ^31^ ^]^ and 2H‐transition metal dichalcogenides.^[^ ^32^ ^]^ A recent noteworthy development by Mu et al. involved the prediction of SOTIs in monolayer (ML) FeSe by utilizing a canted checkboard AFM order, which was supported by both analytical models and first‐principles calculations.^[^ ^33^, ^34^ ^]^ However, the elusive discovery of an intrinsic 2D AFM SOTI remains an ongoing challenge in the realm of high‐order TIs.

In the realm of 2D magnetic materials, recent experiments have successfully synthesized CrSBr, a compound known has a highly anisotropic structure and remarkable air stability.^[^ ^35^, ^36^, ^37^ ^]^ This material features a layered structure, and atomically thin flakes of CrSBr can be obtained through mechanical exfoliation. Interestingly, its magnetism is layer‐dependent. By experimental verification,^[^ ^35^ ^]^ ML CrSBr with a Curie temperature *T*_C_ = 146 K is FM and bilayer (BL) CrSBr with a Néel temperature *T*_N_ = 140 K has A‐type AFM state. Notably, the 2D CrSBr is a semiconductor with a bandgap ≈0.67 eV. Its topological properties have not been carefully investigated before because of its magnetism and trivial bandgap. However, the high‐order topology has not been studied yet.

In this work, we theoretically predict the 2D CrSBr as a realistic example of a magnetic SOTI. More importantly, the SOTI can be realized in its ML and BL structure. For concreteness, its ML structure is a FM SOTI, and its corner states only appear in spin‐up channel, achieving a fully spin‐polarized states. Meanwhile, the SOTI can also exist in its BL structure, with corner states appearing in both spin channels. Moreover, we also discover that these corner states are robust against spin–orbital coupling (SOC) and symmetry breaking perturbations. This characteristic enhances the feasibility of experimentally detecting the magnetic SOTI phase in CrSBr.

## 2 Computational Details

To study the high‐order topological properties of 2D CrSBr, we have performed first‐principles calculations based on the density functional theory (DFT), employing the Vienna ab initio Simulation Package.^[^ ^38^, ^39^, ^40^ ^]^ For the exchange–correlation potential, we adopt the generalized gradient approximation of the Perdew–Burke–Ernzerhof functional.^[^ ^41^ ^]^ The cutoff energy was set as 400 eV. The Brillouin zone (BZ) was sampled by a Monkhorst–Pack *k*‐mesh with size of 9 × 9 × 1. During lattice optimization, energy and force convergence criteria were set as 10^−6^ eV and 0.01 eV Å^−1^, respectively. A vacuum layer with a thickness of 30 Å was taken to avoid artificial interactions between periodic images. To better describe the strong correlation effect of *d* electrons in Cr, the DFT + *U* method was used for calculating the band structures. We have tested the effective *U* value *U*_eff_ = 0–4 eV (see Supplemental Materials) and found the effective *U* value does not affect our conclusion. Then, in our calculation, the common *U* value is set as 4 eV and Hund exchange *J*_H_ = 1 eV. The eigenvalues of the twofold rotation symmetry are obtained by using the IRVSP code.^[^ ^42^ ^]^ The edge states and nanodisk properties are calculated by the Wannier function^[^ ^43^, ^44^, ^45^, ^46^, ^47^ ^]^ and Pybinding method.^[^ ^48^ ^]^ The electronic band structures obtained from the tight‐binding model are in alignment with those derived from DFT (as shown in Figure S4, Supporting Information). Furthermore, our investigation, as illustrated in Figure S6, Supporting Information, involved testing nanodisks of varying sizes. The results indicate that the nanodisk, constructed from the 10 × 10 × 1 supercell, effectively showcases the topological properties of 2D CrSBr while optimizing computational resources.

## 3 Crystal Structure and Magnetism

**Figure 1:**
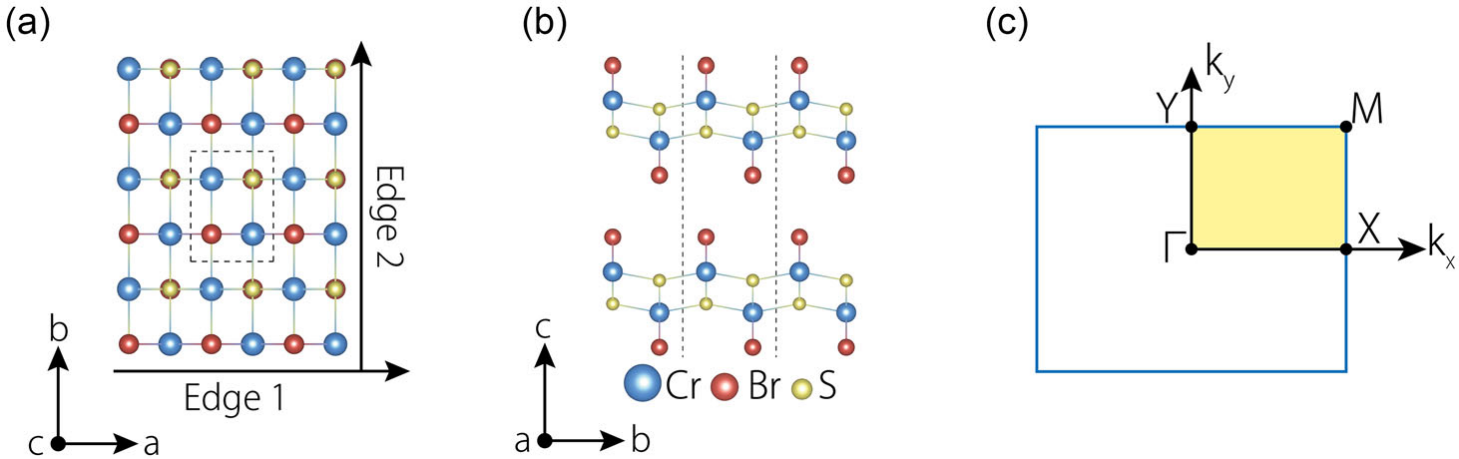
a) Top and b) side views of ML and BL CrSBr; two different edges are given. c) The BZ for 2D CrSBr with high‐symmetry points labeled.

Kihong Lee and co‐workers recently succeeded in synthesizing single crystals of CrSBr using a chemical vapor transport method. They further micromechanically exfoliated *N*‐layered (*N* = 1–6) CrSBr onto the substrates by using scotch tape.^[^ ^35^ ^]^ The crystal structure of ML and BL configurations is shown in **Figure** **1**a,b. Notably, the primitive cell of ML CrSBr comprises six atoms, and the BL CrSBr consists of 12 atoms, as indicated within the dashed rectangle in Figure 1a,b. Because BL CrSBr is essentially composed of two ML structures arranged in an A–A stacking fashion (as shown in Figure 1b), both ML and BL CrSBr share the same space group, *Pmmn* (No.59), generated by three rotation symmetries along the *x*, *y*, and *z* axes (C_2*x*_, C_2*y*_, C_2*z*_). A tetragonal finite‐size nanodisk with a 3 × 3 × 1 supercell of ML and BL CrSBr is constructed as shown in Figure 1a, in which one can observe that there are two different edges, denoted as “Edge 1” and “Edge 2”. The structural parameters are consisted with previous work (see Table S1, Supporting Information).^[^ ^49^, ^50^ ^]^ The corresponding BZ is shown in Figure 1c.

It is worth noting that this van der Waals semiconductor manifests intriguing magnetic properties in its low‐layer structure. Previous experiments and calculations have confirmed that ML CrSBr exhibits ferromagnetism, while BL CrSBr adopts an A‐type AFM state, in which two FM MLs couple antiferromagnetically along the stacking direction (see Figure S1, Supporting Information). Furthermore, both ML and BL CrSBr possess an easy magnetization axis along the *b‐*axis.^[^ ^35^, ^36^, ^37^, ^49^, ^50^ ^]^ We provide the PDOS of ML and BL CrSBr in Figure S2 and S3, Supporting Information, in which one can see that the energy bands near the Fermi level are largely contributed by the Cr *d*, S *p*, and Br *p* orbitals. Importantly, it shows semiconductive behaviors. In the following, we will study the topology of its gapped bulk.

## 4 SOTI in ML CrSBr

**Figure 2:**
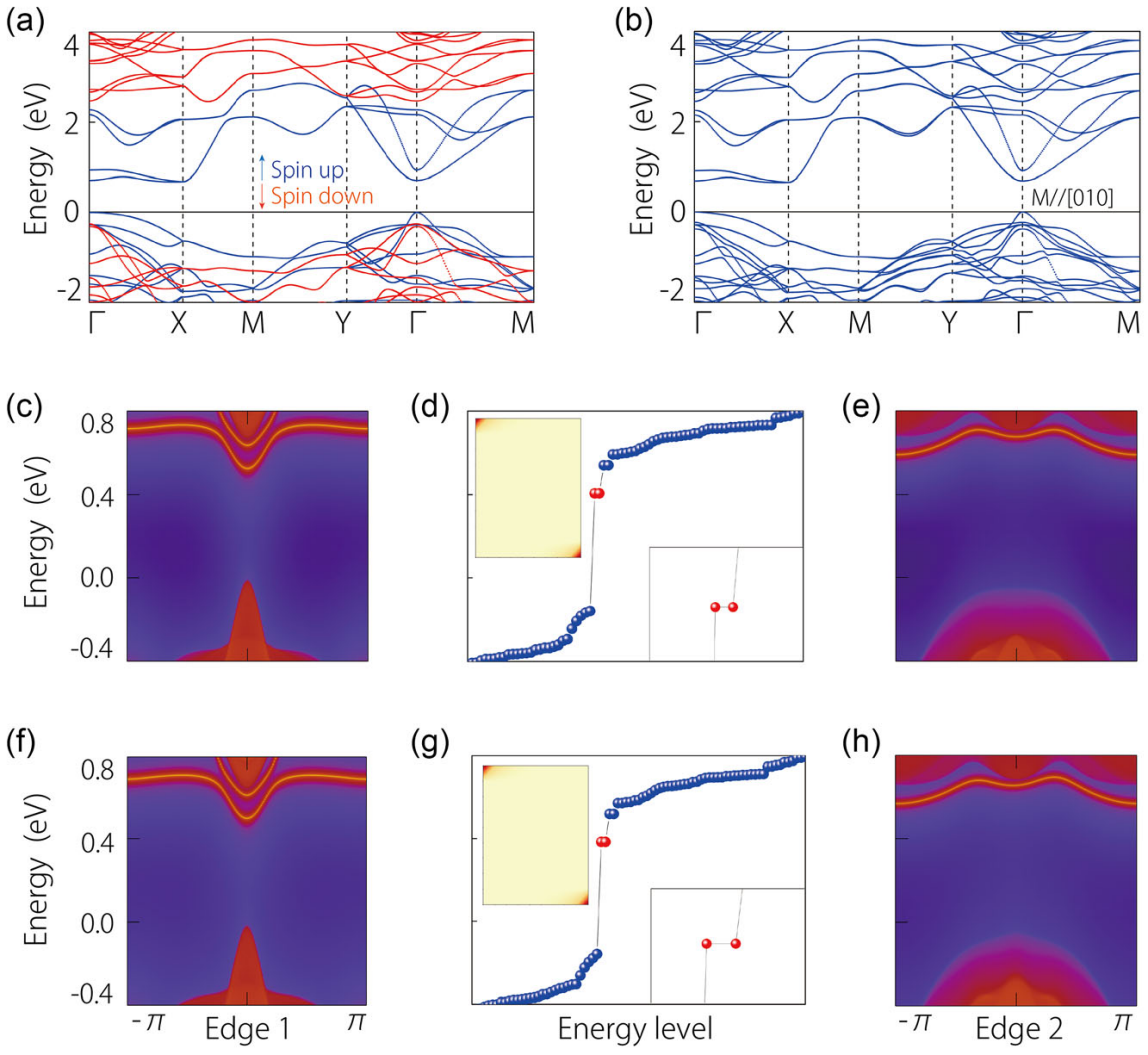
Electronic band structures for ML CrSBr a) without and b) with SOC. c,e) The projected spectra in the absence of SOC for Edge 1 and Edge 2, respectively. f,h) The projected spectra with SOC for Edge 1 and Edge 2, respectively. d,g) The energy spectrum of a rectangle nanodisk without/with SOC, respectively. Their corresponding charge distribution of the corner modes is illustrated in the inset figures, which are localized at corners.

Let us first study topological property of ML CrSBr. As shown in **Figure** **2**a, we plot the electronic band structure for the spin‐up and spin‐down states in the absence of SOC. We find that ML CrSBr is a FM semiconductor with a large bandgap of ≈0.67 eV. Without considering SOC, the spin‐up and spin‐down subspaces are decoupled (respectively marked with blue and red lines in Figure 2a), we can consider each spin channel as a spinless system. Therefore, all spatial symmetries in the space group *Pmmn* and time‐reversal symmetry (*T*) are preserved for each spin channel.

Due to the presence of C_2*z*_ and *T*, we can calculate the topological index and fractional corner charge, which is determined by the C_2*z*_‐symmetric eigenvalues of the occupied states at high‐symmetry points in BZ, to characterize the topology of the ML CrSBr.^[^ ^51^ ^]^ Specifically, the C_2*z*_‐symmetric eigenvalues at the high‐symmetry points $\Pi_{p}^{\left(2\right)}$ of the BZ are denoted as

(1) $$\Pi_{p}^{\left(2\right)}=e^{\pi i(p-1)},\;p=1,\;2$$

Here, the superscript “2” labels the twofold rotation symmetry. Correspondingly, the integer topological invariants can be obtained through C_2*z*_‐symmetric eigenvalues at $\Pi_{p}^{\left(2\right)}$ compared to a certain reference point Γ = (0, 0)

(2) $$\left[\Pi_{p}^{\left(2\right)}\right]\equiv \#\Pi_{p}^{\left(2\right)}-\#\Gamma_{p}^{\left(2\right)}$$

where $\#\Pi_{p}^{\left(2\right)}$is the number of occupied states with eigenvalue $\Pi_{p}^{\left(2\right)}$. Therefore, the topological indices $\chi^{\left(2\right)}$ read

(3) $$\chi^{\left(2\right)}=(\left[X_{1}^{\left(2\right)}\right],\;\left[Y_{1}^{\left(2\right)}\right],\;\left[M_{1}^{\left(2\right)}\right]$$

Then, the fractional corner charge $Q_{c}^{\left(2\right)}$ associated with T and C_2*z*_ symmetries is given by

(4) $$Q_{c}^{\left(2\right)}=\frac{e}{4}(-\left[X_{1}^{\left(2\right)}\right]-\left[Y_{1}^{\left(2\right)}\right]+[M_{1}^{\left(2\right)}])\text{mod}\;e$$

where *e* is the charge of a free electron.

Through first‐principles calculations, we find that ML CrSBr has 22 occupied states in the spin‐up channel and 16 occupied states in the spin‐down channel. Through screening and calculations, we obtained the topological index $\chi^{\left(2\right)}$ and corner charge $Q_{c}^{\left(2\right)}$, as shown in **Table** **1**. The results indicate that the spin‐up channel of ML CrSBr possesses a nonzero fractional corner charge $Q_{c,\;↑}^{\left(2\right)}$, which is equal to *e*/2. Conversely, the corner charge in spin‐down channel is equal to 0. That is, ML CrSBr is a SOTI, carrying a nonzero fractional corner charge in one spin channel, resulting in a fully spin‐polarized high‐order phase. In addition, we also get the same result for different effective values of *U* (as detailed in Table S2, Supporting Information), so that the *U* value does not affect its topological phase.

**Table 1:** Corner charges of ML CrSBr under *U*_eff_ = 3 eV in the spin‐up and spin‐down channel

System	$\#\Gamma_{1}^{\left(2\right)}$	$\#X_{1}^{\left(2\right)}$	$\#M_{1}^{\left(2\right)}$	$\#Y_{1}^{\left(2\right)}$	$\left[X_{1}^{\left(2\right)}\right]$	$\left[M_{1}^{\left(2\right)}\right]$	$\left[Y_{1}^{\left(2\right)}\right]$	$Q_{c,\;↑}^{\left(2\right)}$	$Q_{c,\;↓}^{\left(2\right)}$
Spin up	12	11	12	11	‐1	0	‐1	$\frac{e}{2}$	–
Spin down	8	8	8	8	0	0	0	–	0

As we all know, the hallmark of the *n*th high‐order topological phase is the presence of surface states at (*d–n*) boundary. We then calculate the corner states of the ML CrSBr. Before checking the presence of corner states, we first confirm the gap in edge states along different boundaries. As shown in Figure 2c,e, both spectra of edge states along Edge 1/2 show an energy gap. To confirm the presence of corner states, we construct a tetragonal finite‐size nanodisk with a 10 × 10 × 1 supercell. As aforementioned, the C_2*z*_ symmetry is closely associated with the corner charge, such that the nanodisks we constructed preserve this symmetry. Indeed, as shown in Figure 2d, there are a pair of local electron states existing in the gap. We further confirm their wave function distribution in the nanodisk. As shown in the upper left illustration in Figure 2d, the electrons are indeed localized at the corners of the nanodisk. The results show that ML CrSBr is a 2D SOTI with fully spin‐polarized corner states.

Next, we delve into the effects of SOC. The resultant electronic band structure, which includes SOC, is presented in Figure 2b. Notably, it is evident that SOC exerts a relatively weak impact on the electronic band structure by comparing cases with/without SOC. In essence, the electronic band structure with SOC can be considered as a superposition of spin‐up and spin‐down states in the absence of SOC. Akin to case without SOC, even with the inclusion of SOC, the edge states at Edge 1 and Edge 2 retain a gap across the entire BZ, as depicted in Figure 2f,h. Crucially, the corner states persist within the gap, as depicted in Figure 2g. Intriguingly, despite the presence of SOC, a pair of corner states still exist. This implies that in the scenario with SOC, the nonzero corner charge continues to be predominantly contributed by states within the spin‐up channel. Hence, the corner states also maintain complete spin polarization. Consequently, ML CrSBr exhibits the characteristics of a 2D magnetic SOTI with fully spin‐polarized corner states, both in the presence and absence of SOC. Such a feature is advantageous for detecting these corner states through spin‐resolved scanning tunneling spectroscopy (STS) techniques.

Given the van der Waals coupling between layers, what will happen when two MLs stacking? We will discuss the topological phase in the following.

## 5 SOTI in BL CrSBr

**Figure 3:**
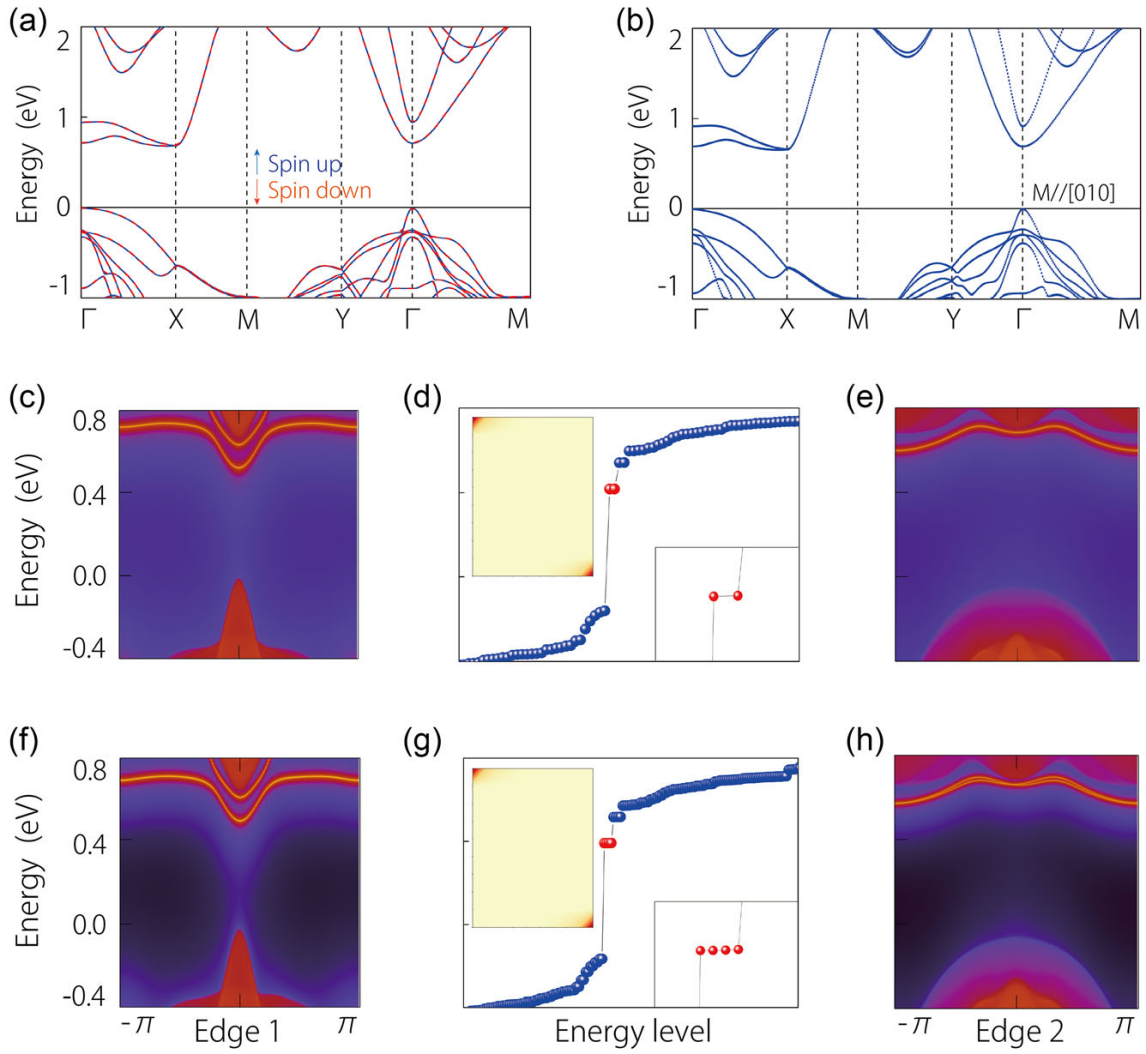
Electronic band structures for BL a) (without SOC) and b) (with SOC) CrSBr. The projected spectra c,f) for Edge 1 and e,h) for Edge 2 of BL CrSBr without/with SOC, respectively. d,g) Their energy levels without/with SOC are plotted, respectively. The upper inset figures show the charge distribution of the rectangle nanodisk, and lower inset figures indicate the enlarged views for the corner states.

It has been demonstrated that BL CrSBr exhibits a collinear A‐type AFM configuration. As a result, the spin‐up and spin‐down states still remain decoupled, enabling each spin channel to be treated as a spinless system while retaining time‐reversal symmetry. Without considering SOC, the electronic band structure of BL CrSBr is plotted in **Figure** **3**a, the spin up and spin down, respectively, marked by blue and red lines. The band structure in the low‐energy region shown in Figure 3a closely resembles that of the spin‐up states in ML CrSBr. Consequently, it is reasonable to infer that the SOTI phase is preserved in BL CrSBr.

To ascertain this hypothesis, we have employed a tetragonal nanodisk model created with a 10 × 10 × 1 supercell to demonstrate the presence of 0D corner states. In the absence of SOC, the edge spectra for BL CrSBr within the spin‐up channel are exhibited in Figure 3c,e. These spectra distinctly reveal a gap in the edge states along two distinct boundaries. We then computed the discrete spectrum for this nanodisk, as shown in Figure 3d. Notably, a pair of localized states emerges within the energy gap. To further substantiate this finding, we examine the spatial distribution of the wave functions within the nanodisk. Akin to the corner states in ML, the localized states are confined to two opposing corners. This provides robust evidence for the presence of corner states. Moreover, due to the decoupling of the spin‐up and spin‐down channels within BL CrSBr without SOC, the spin‐down channels are expected to host high‐order topological properties as the spin‐up channel. Hence, in the absence of SOC, BL CrSBr qualifies as an AFM SOTI and characterized by a pair of 0D corner states in each spin channel.

Upon including SOC, with the magnetic moment oriented along the *b*‐axis, the electronic band structure is plotted in Figure 3b. In a manner akin to the ML structure, BL CrSBr also has weak SOC. The band structure of BL CrSBr with SOC remains largely unaltered compared to that without SOC, maintaining a significant gap of ≈0.67 eV. Thus, when SOC is considered, the BL CrSBr preserves the second‐order topological properties of each spin channel. Furthermore, the gaps within the states along Edge 1 and Edge 2 still exist, as illustrated in Figure 3f,h. Next, the energy spectrum for the nanodisk is plotted. In contrast to the ML case, both spin channels now contribute a pair of localized states. Consequently, two pairs of localized states are expected to emerge in the gap due to the presence of SOC. As depicted in Figure 3g, four local states indeed appear within the gap, with their spatial distribution centered at the corners of the nanodisk. Consequently, it is clear that even in the presence of SOC, the synthesized AFM BL CrSBr maintains its status as a SOTI, substantiated by the existence of corner states.

In contrast to ML CrSBr, the BL structure maintains an AFM ground state. However, the BL configuration still manifests a SOTI phase, both in the presence and absence of SOC. Given the similarities between ML and BL cases, the SOTI phase appears to be closely associated with the twofold rotation symmetry C_2*z*_.

## 6 Robustness of Corner States

**Figure 4:**
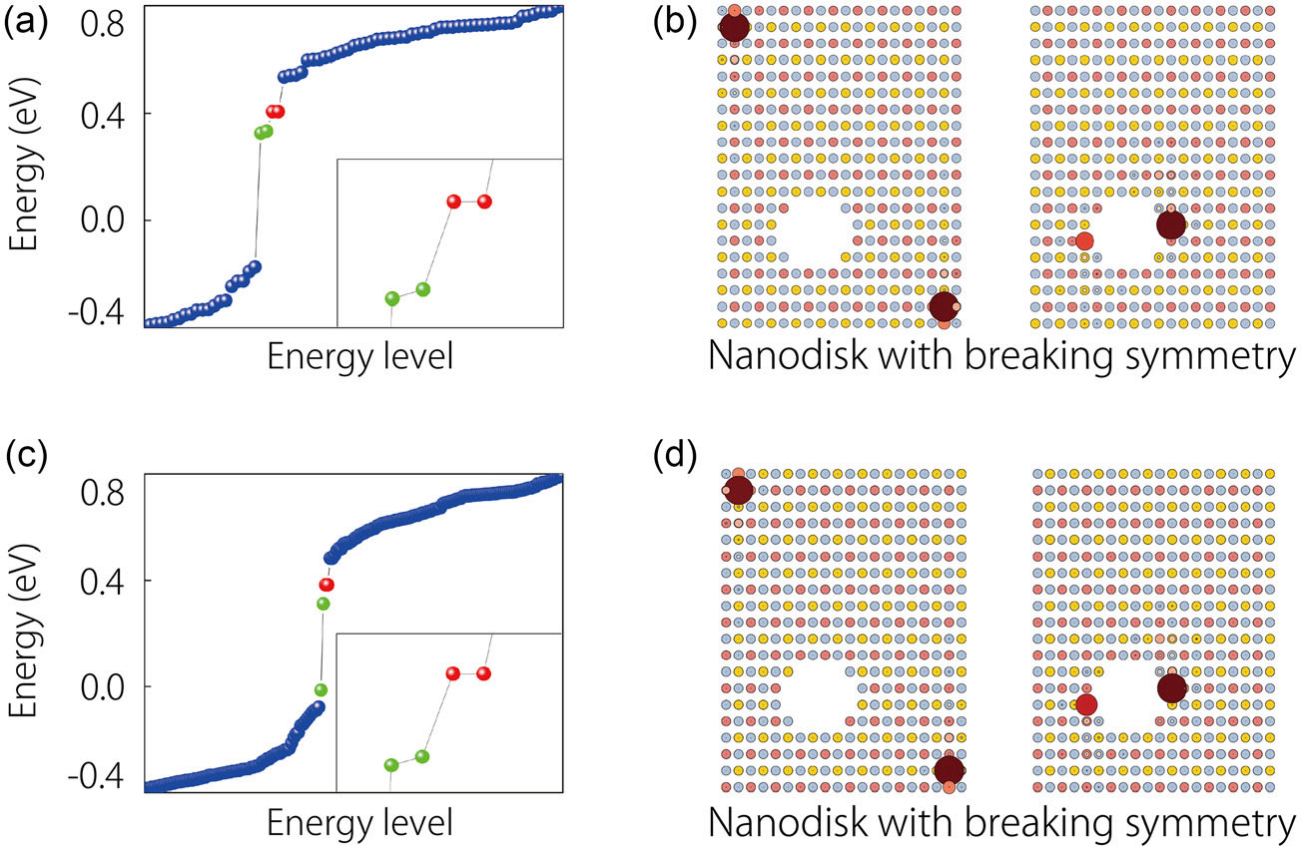
Corner states in ML and BL CrSBr under symmetry breaking perturbations. a,c) The energy spectrum of the nanodisk in ML and BL CrSBr with breaking C_2*z*_ symmetry, respectively. Red dots refer to the corner states; green dots denote as accidentally appearing states. b,d) Corner states are still located at corners of the sample, and accidentally appearing state is pinned at artificial distortion.

**Figure 5:**
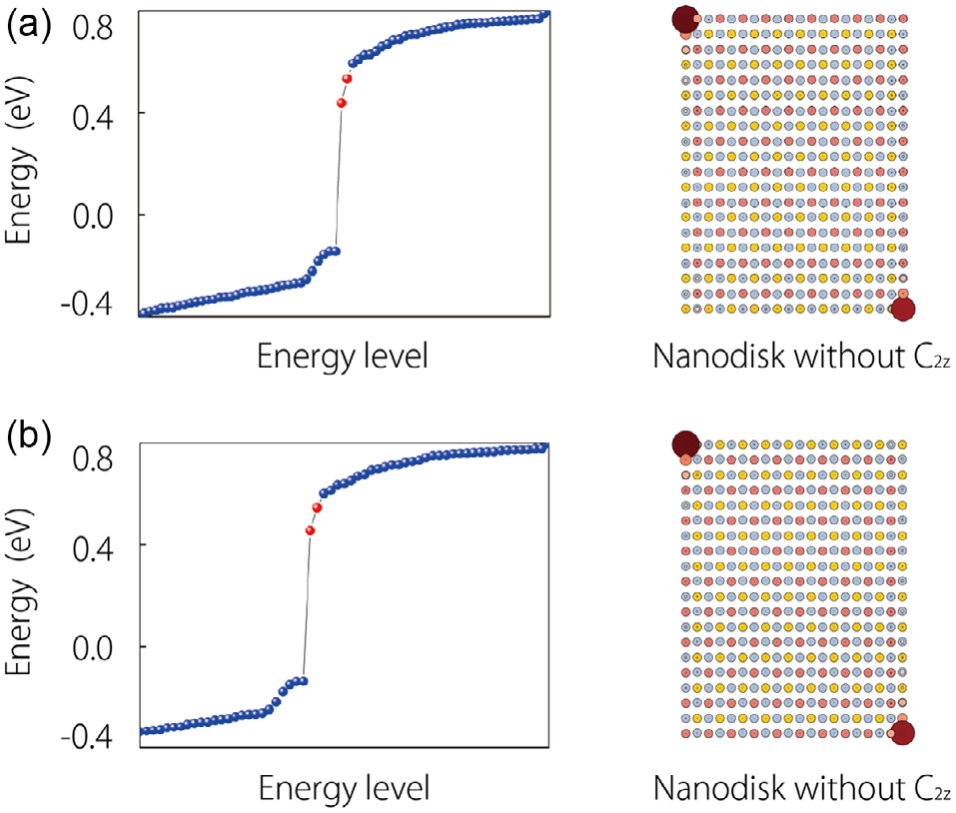
Without considering SOC, the energy spectrum and corner states of the nanodisk in a) ML CrSBr and b) BL CrSBr after moving Cr atoms.

As established in previous studies,^[^ ^52^, ^53^, ^54^ ^]^ we can affirm that the topological corner states within ML and BL CrSBr remain robust even in the presence of symmetry‐breaking perturbations. To demonstrate this resilience, we introduce significant artificial perturbation within the tetragonal nanodisks, thereby breaking the C_2*z*_ symmetry, as illustrated in **Figure** **4**b,d. Subsequently, we reevaluate the electronic localization properties of these perturbed nanodisks in the absence of SOC, as depicted in Figure 4a,c. In both cases, it is evident that localized states are still present. Analyzing their spatial distribution, we find that the original corner states still exist, maintaining their position at the corners of the nanodisks. Additionally, a few discrete states, denoted by green dots in Figure 4a,c, appear near the artificial gaps. However, these corner states surrounding the artificial holes do not affect the energy or position of the topological corner states, meaning that they do not compromise the SOTI characteristics of ML and BL CrSBr. Furthermore, we intentionally break the C_2*z*_ symmetry by displacing atoms. Specifically, we shift the Cr atom in the upper left corner of the primitive cell of ML and BL CrSBr by 0.1 Å along the *b*‐axis, as illustrated in Figure S5, Supporting Information. Using these modified primitive cells, we construct finite‐size nanodisks composed of a 10 × 10 × 1 supercell. The calculation results, presented in **Figure** **5**, indicate that the corner state still exists, but the energy degeneracy of the corner state is disrupted due to the displacement of the Cr atom, which significantly breaks the C_2*z*_ symmetry. Consequently, the displacement of atoms does not result in the creation of dangling bonds that could give rise to additional localized electron states. This resilience enhances the feasibility of their experimental detection.

## 7 Summary

In summary, our theoretical investigation has unveiled the presence of magnetic SOTI states in both ML and BL CrSBr. An intriguing aspect of this layered material is that its magnetic configuration is layer‐dependent, with ML CrSBr exhibiting a FM ground state, while BL CrSBr adopts an AFM ground state. Remarkably, both configurations harbor a SOTI phase, with FM (AFM) ML (BL) CrSBr showcasing a SOTI phase with and without SOC. The corner states in FM ML CrSBr exclusively appear in the spin‐up channel, featuring fully spin‐polarized corner states. This characteristic makes it feasible to detect these corner states through STS measurements. Conversely, AFM BL CrSBr boasts a pair of 0D corner states in both spin channels, even in the presence or absence of SOC. This represents the first concrete example of AFM SOTIs in 2D materials.

Furthermore, these corner states within ML and BL CrSBr exhibit a remarkable resistance to SOC and remain robust against symmetry‐breaking perturbations, enhancing their feasibility for experimental detection. In summation, our findings contribute to the growing family of 2D magnetic SOTIs, encompassing both FM and AFM variants. This provides a promising platform for delving into the interplay between high‐order topological phases and magnetism.

## Conflict of Interest

The authors declare no conflict of interest.

## Supporting information

Supplementary Material

## Data Availability

The data that support the findings of this study are available from the corresponding author upon reasonable request.
