# Supplementary material for: Second‐Order Topological Insulator in Ferromagnetic Monolayer and Antiferromagnetic Bilayer CrSBr
Source: Small Sci. 2024 Mar 15;4(6):2300356. doi: 10.1002/smsc.202300356 (PMC11935174; doi:10.1002/smsc.202300356)
Supplement: Supplementary file 1 — Supplementary Material [file SMSC-4-2300356-s001.pdf]

Supplementary Information for

Second-Order Topological Insulator in ferromagnetic monolayer and antiferromagnetic bilayer CrSBr

Zhenzhou Guo[1,2], Haoqian Jiang[1], Lei Jin[1], Xiaoming Zhang[1], Guodong Liu[1], Ying Liu[1,*], and Xiaotian Wang [2,*]

[1] *State Key Laboratory of Reliability and Intelligence of Electrical Equipment, and School of Materials Science and Engineering, Hebei University of Technology, Tianjin 300130, China.*

[2] *Institute for Superconducting and Electronic Materials (ISEM), University of Wollongong, Wollongong 2500, Australia.*

[*] E-mail of corresponding author: ying_liu@hebut.edu.cn; xiaotianw@uow.edu.au

I. Structural parameters

Through first-principles calculations, we obtained fully relaxed cells for monolayer (ML) and bilayer (BL) CrSBr. The lattice constants and bond lengths are shown in the table below.

Table S1. The structural parameters of ML and BL CrSBr, including the lattice constants (a, b), the bond lengths of Cr-Br (d_1) and Cr-S (d_2), the distance layer (d_3) defined by the smallest difference between the z coordinates of Br atoms in different layers.

System	*a (Å)*	*b (Å)*	d_1 *(Å)*	d_2 *(Å)*	d_3 *(Å)*
ML	3.546	4.735	2.523	2.414	-
BL	3.545	4.735	2.524	2.414	3.309

II. Magnetic ground state

We have plotted the schematic diagrams of magnetic ground states of ML and BL CrSBr. As shown in Figure S1, ML CrSBr exhibits ferromagnetism. However, for the BL structure of two-dimensional CrSBr, although each layer is ferromagnetic, there is antiferromagnetic coupling between the two layers, forming an A-type antiferromagnetic ground state.

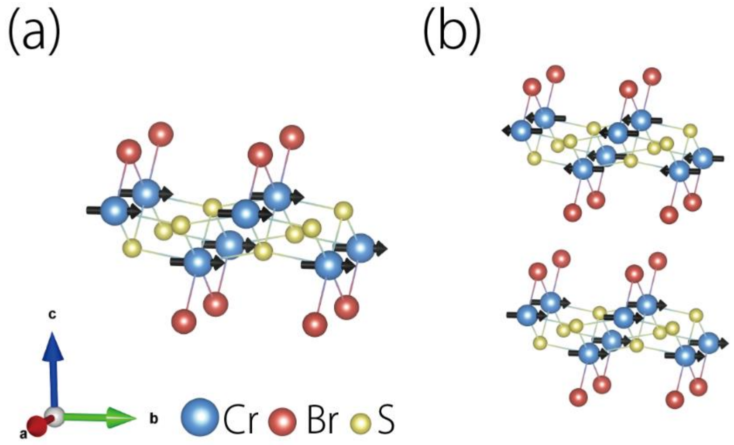

Figure S1. Schematic diagrams of magnetic ground states of (a) ML and (b) BL CrSBr.

III. Projected density of states of ML and BL CrSBr

We have plotted the Projected Density of States (PDOS) of ML and BL CrSBr. As depicted in Fig. S1, when the SOC is not considered, the low-energy states ML and BL CrSBr in both spin channels mainly originate from the Cr-d, Br-p, and S-p orbitals.

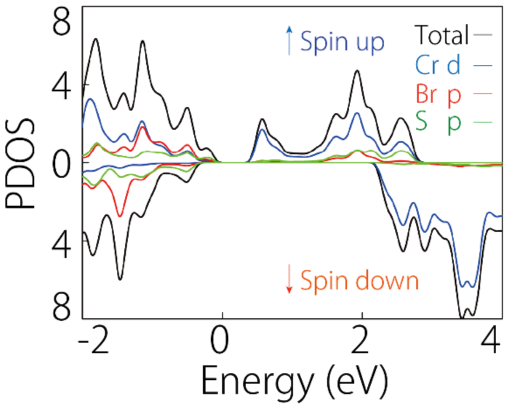

Figure S2. PDOS of ML CrSBr in the spin-up and spin-down channels.

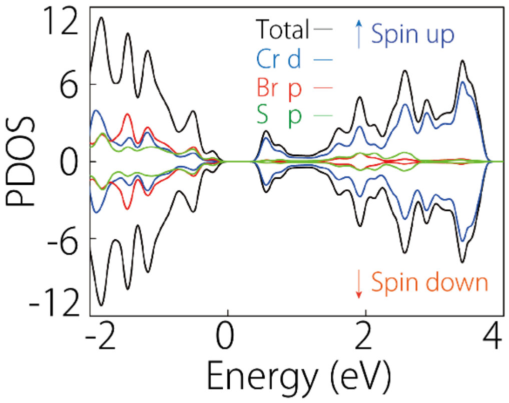

Figure S3. PDOS of BL CrSBr in the spin-up and spin-down channels.

IV. Fractional corner charges of ML and BL CrSBr under different *U* values

We have calculated the fractional corner charge of ML CrSBr at different *U* values and found that it remains e/2 in the spin-up channel and zero in the spin-down channel, as shown in Table S2. This means that the conclusion that ML CrSBr is a HOTI is not affected by *U* values.

Table S2. Fractional corner charges of ML CrSBr under different U_{eff} values without considering SOC.

U_{eff} values	Spin up $\#\Gamma_1^{(2)}$	$\#X_1^{(2)}$	$\#M_1^{(}$[illegible]	$\#Y_1^{(2)}$	$Q_c^{(2)}$	Spin down $\#\Gamma_1^{(2)}$	$\#X_1^{(2)}$	$\#M_1^{(2)}$	$\#Y_1^{(2)}$	$Q_c^{(2)}$
0	12	11	12	11	$\frac{e}{2}$	8	8	8	8	0
1	12	11	12	11	$\frac{e}{2}$	8	8	8	8	0
2	12	11	12	11	$\frac{e}{2}$	8	8	8	8	0
3	12	11	12	11	$\frac{e}{2}$	8	8	8	8	0
4	12	11	12	11	$\frac{e}{2}$	8	8	8	8	0

V. The comparison between band structures of maximally localized Wannier functions and DFT results.

We have consistent the body band of TB model with that obtained by DFT. As shown in Figure S4, the obtained tight-binding (TB) model fits the DFT band structure well. This indicates that the TB model we constructed is consistent with the material properties, and the subsequent results can also fully reflect the actual situation of two-dimensional CrSBr.

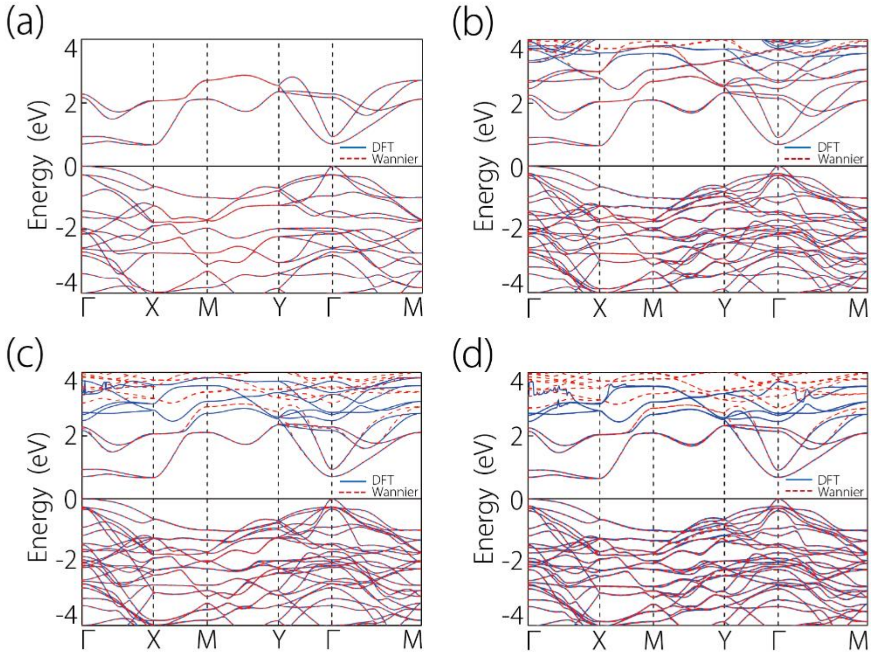

Figure S4. The comparison between band structures of maximally localized Wannier

functions and DFT results. Electronic band structures for (a) ML CrSBr and (c) BL CrSBr in the spin-up channel. Electronic band structures for (b) ML CrSBr and (d) BL CrSBr with SOC.

VI. Breaking symmetry by moving Cr atoms

To break the C_{2z} symmetry in 2D CrSBr, we moved the Cr atom marked with A five-pointed star of the primitive cell of ML and BL CrSBr by 0.1 Å along the b-axis (see in Figure S5).

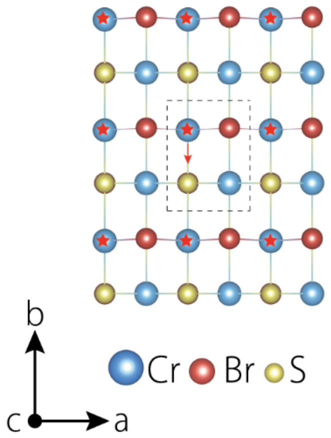

Figure S5. An artificial distortion that breaks the C_{2z}. Each Cr atom marked with A five-pointed star is displaced from its equilibrium position by 0.1 Å.

VII. Energy spectrums of finite-sized nanodisks under different size

We calculate energy spectrums and corner states in spin-up channel using different tetragonal finite-size nanodisks of ML and BL CrSBr. The result is shown in Figure S6. These finite-sized nanodisks composed of 5×5×1,10×10×1 and 15×15×1 supercells of ML and BL CrSBr all obtained the corner states of energy degeneracy at ~0.4 eV. Therefore, the finite-size nanodisks composed of 10*10*1 supercells adopted by us can well reflect the high-order topological properties of ML and BL CrSBr. It is worth mentioning that if the size of the nanodisk is smaller than 5*5*1 supercell, the result may be non-degenerate corner states.

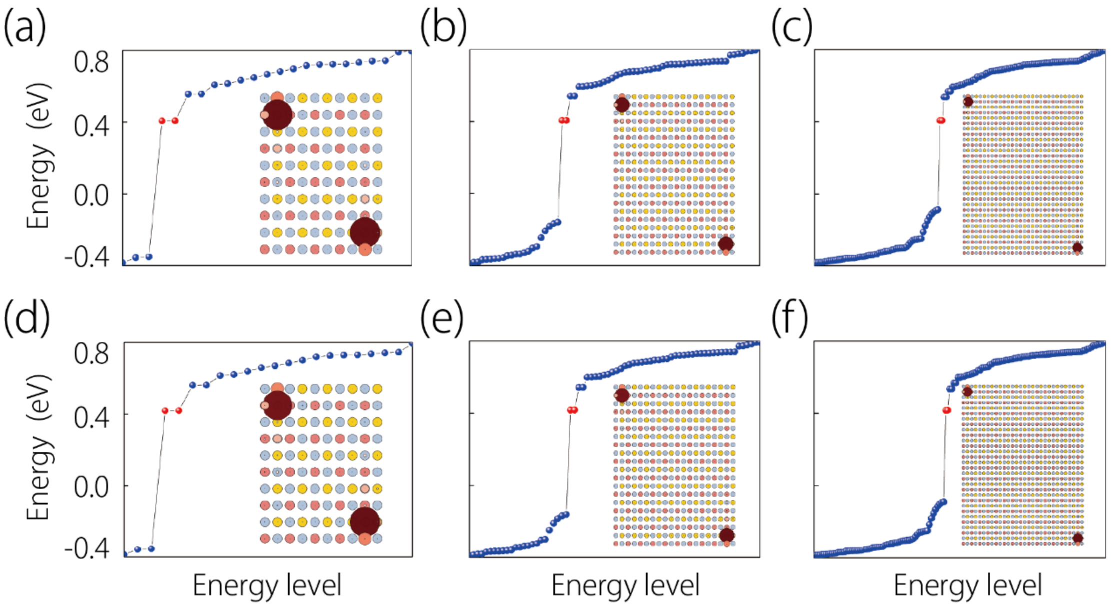

Figure S6. In the absence of SOC, the energy spectrums of nanodisks in the spin-up channel for ML (a-c) and BL (d-f) CrSBr. (a) 5×5×1, (b) 10×10×1 and (c) 15×15×1 supercells of ML CrSB; (d) 5×5×1, (e) 10×10×1 and (f) 15×15×1 supercells of BL CrSBr.
